# Multidisciplinary Telemedicine in Healthcare During and After the COVID-19 Pandemic: A Narrative Review

**DOI:** 10.3390/life15050783

**Published:** 2025-05-14

**Authors:** Angelica Gherman, Diana Andrei, Călin Marius Popoiu, Emil Robert Stoicescu, Mihaela Codrina Levai, Isabella Ionela Stoian, Vlad Bloancă

**Affiliations:** 1 Ph.D. School Department, “Victor Babeş” University of Medicine and Pharmacy of Timisoara, 300041 Timisoara, Romania; angelica.gherman@umft.ro; 2 Research Center for Medical Communication, “Victor Babeş” University of Medicine and Pharmacy of Timisoara, Eftimie Murgu Square No. 2, 300041 Timisoara, Romania; stoicescu.emil@umft.ro (E.R.S.); codrinalevai@umft.ro (M.C.L.); 3 Department of Balneology, Medical Rehabilitation and Rheumatology, “Victor Babeş” University of Medicine and Pharmacy of Timisoara, 300041 Timisoara, Romania; 4 Department of Pediatric Surgery, “Victor Babeş” University of Medicine and Pharmacy of Timisoara, 300041 Timisoara, Romania; 5 Radiology and Medical Imaging University Clinic, “Victor Babeş” University of Medicine and Pharmacy of Timisoara, Eftimie Murgu Square No. 2, 300041 Timisoara, Romania; 6 Research Center for Pharmaco-Toxicological Evaluations, “Victor Babeş” University of Medicine and Pharmacy of Timisoara, Eftimie Murgu Square No. 2, 300041 Timisoara, Romania; 7 Field of Applied Engineering Sciences, Specialization Statistical Methods and Techniques in Health and Clinical Research, Faculty of Mechanics, “Politehnica” University Timisoara, Mihai Viteazul Boulevard No. 1, 300222 Timisoara, Romania; 8 Department of Microscopic Morphology, “Victor Babeş” University of Medicine and Pharmacy of Timisoara, 300041 Timisoara, Romania; isabella.stoian@umft.ro; 9 Department of Plastic Surgery, “Victor Babeş” University of Medicine and Pharmacy of Timisoara, Eftimie Murgu Square 2, 300041 Timisoara, Romania; bloanca.vlad@umft.ro

**Keywords:** COVID-19 pandemic, multidisciplinary teams, virtual consultations, virtual multidisciplinary team, virtual multidisciplinary board, telemedicine, post-pandemic care

## Abstract

The COVID-19 pandemic accelerated the adoption of virtual multidisciplinary teams (MDTs), transforming healthcare delivery through telemedicine. This review examines the integration of telemedicine into multidisciplinary care across various medical specialties, highlighting its benefits and challenges. A comprehensive literature search was conducted across PubMed, Google Scholar, Scopus, and Web of Science, using keywords related to telemedicine and MDTs. Inclusion criteria focused on studies discussing telemedicine implementation in multidisciplinary care, as well as its effectiveness and impact on patient outcomes, while non-English studies, single-case reports, and articles lacking explicit discussions on MDT integration were excluded. Data extraction covered telemedicine platforms, specialties involved, patient satisfaction, and clinical outcomes. Our findings suggest that virtual MDTs enhance efficiency, accessibility, and patient satisfaction, particularly in remote and underserved areas. However, challenges, such as technological barriers, disparities in digital access, and maintaining effective team communication, persist. Despite these limitations, telemedicine has demonstrated significant potential in improving diagnostic accuracy and treatment coordination. Future efforts should focus on optimizing infrastructure, digital training for healthcare providers, and regulatory frameworks to guarantee long-term sustainability.

## 1. Introduction

The COVID-19 pandemic has dramatically transformed healthcare delivery worldwide, accelerating the adoption of telemedicine across various medical specialties. With the unprecedented challenges posed by the pandemic, healthcare systems had to rapidly adapt to ensure continued patient care while minimizing the risk of viral transmission [1,2]. Millions of patients who were unable to receive traditional in-person care were able to continue receiving it through telemedicine, which became an invaluable resource in this situation [3]. The integration of telemedicine into multidisciplinary care models became not just a necessity but a transformative opportunity to reimagine healthcare delivery. Telemedicine is a broad concept that includes various technologies and services, allowing healthcare professionals to deliver care remotely. It encompasses digital applications, audio and video online consultations, remote monitoring, patient guidance, and electronic health records [4,5]. Telemedicine specifically involves remote clinical care, whereas telehealth encompasses a broader range of remote activities, including healthcare provider training, continued medical/public health education, administrative virtual boards, and electronic data sharing [6,7].

Multidisciplinary care, which brings together professionals from diverse fields, such as medicine, nursing, psychology, social work, and rehabilitation [8], has long been recognized as a gold standard for managing complex health conditions [9]. However, the pandemic forced these teams to adapt rapidly to remote collaboration, leveraging telemedicine platforms to coordinate care, share expertise, and support patients in new ways [10]. This shift was not without challenges, including technological barriers, disparities in access to digital tools, and the need to maintain the human connection that is central to effective care. However, it also revealed the potential for telemedicine to enhance the efficiency, reach, and inclusivity of multidisciplinary teams [11]. In order to manage complex situations, especially for patients with comorbidities, infectious diseases, and chronic illnesses requiring long-term care, the COVID-19 crisis highlighted the necessity of collaboration among specialists [10,12]. In this context, virtual multidisciplinary team meetings became essential, allowing healthcare professionals to collaborate remotely, review complex cases, and develop comprehensive patient management strategies. Telemedicine platforms facilitated efficient communication between specialists, primary care providers, and allied healthcare professionals, ensuring that patients received coordinated and rapid care [13].

Multidisciplinary virtual consultations offer significant advantages in patient care. These consultations improve patient satisfaction by reducing the logistical burden associated with in-person visits, including travel time and other associated costs [14,15]. Additionally, they make sure that patients receive comprehensive evaluations in a single appointment, where they can engage with multiple healthcare professionals simultaneously. This collaborative approach not only facilitates a more thorough assessment of the patient’s condition but also enhances diagnostic accuracy and treatment planning by incorporating diverse medical perspectives [9]. Furthermore, virtual multidisciplinary care is especially beneficial for patients residing in remote or underserved areas who may face challenges in accessing specialized healthcare services [16].

Before the COVID-19 pandemic, telecare was largely experimental. However, it has since become an essential tool, demonstrating its effectiveness in assessing, managing and treating various diseases and guaranteeing patient follow-up [13]. This narrative review explores the connection between multidisciplinary care and telemedicine across medical specialties during the COVID-19 era. It examines the strategies that healthcare systems employed to integrate telemedicine into multidisciplinary care, the outcomes achieved, and the lessons learned. We also consider patient/healthcare provider satisfaction and the recommendations of different organizations worldwide. This article reviews recent experiences to guide healthcare systems in improving and expanding the innovations introduced during the COVID-19 era, aiming for more effective and accessible care models.

## 2. Materials and Methods

### 2.1. Literature Search Strategy

A comprehensive literature search was conducted by two independent authors using multiple scientific databases, including PubMed, Google Scholar, Scopus, and Web of Science. The search strategy utilized keywords, such as “telemedicine”, “multidisciplinary care”, “COVID-19 pandemic” “virtual consultations”, and “multidisciplinary team meetings”. Boolean operators (“AND”, “OR”) were used to refine the search. Additionally, article titles and abstracts containing synonyms of the search terms were reviewed. The initial selection process involved screening titles and abstracts for eligibility. To maintain high-quality evidence, only peer-reviewed articles, systematic reviews, and meta-analyses were considered. From the searched criteria, articles published between 2020 and 2024 (a 5-year period) were included in the review, as telemedicine has been increasingly implemented and used since the start of COVID-19 pandemic.

### 2.2. Eligibility Criteria

The inclusion criteria for article selection comprised studies discussing the implementation of telemedicine in multidisciplinary care, its effectiveness across various medical specialties, and its impact on patient outcomes and healthcare efficiency. Exclusion criteria included non-English studies, single-case reports, and articles that did not explicitly address the integration of telemedicine into multidisciplinary healthcare settings. Given the large number of articles on telemedicine and multidisciplinary care in telemedicine, we conducted a critical evaluation to select the articles that explicitly described the composition and roles of the multidisciplinary teams, their specific interventions, and the key medical specialties involved. Our focus was on studies that provided detailed insights into how these teams functioned and contributed to patient management.

### 2.3. Data Extraction

Data extraction focused on key parameters, such as the type of telemedicine platform utilized, the specialties involved, patient satisfaction, clinical outcomes, and healthcare provider perspectives. A narrative synthesis approach was employed to analyze and summarize findings from different studies. No statistical analysis was performed, given the type of the review and the lack of standardized methods to evaluate telemedicine. In order to review best practices, legal issues, and potential future directions for telemedicine in multidisciplinary settings, professional medical organizations’ consensus statements and expert viewpoints were also examined. A thorough and impartial evaluation of telemedicine’s contribution to improving multidisciplinary care both during and after the COVID-19 pandemic was guaranteed by the methodological approach.

## 3. General Considerations

Telemedicine has evolved as a revolutionary alternative to healthcare delivery, especially in multidisciplinary care [17]. It has been utilized to reduce in-person COVID-19 exposure while preserving healthcare capacity for individuals who require in-person treatment the most. Many health centers immediately switched to telemedicine visits instead of in-person visits during the pandemic [18,19]. While it was recognized for its potential to address physician shortages and improve patient access and convenience well before the pandemic, its adoption had been relatively moderate prior to the crisis [12,20]. During the COVID-19 pandemic, regulatory waivers and expanded reimbursement facilitated a surge in tele-multidisciplinary collaboration, driving down no-show rates and inpatient transfer costs, while maintaining, or even improving, clinical outcomes under crisis conditions. This real-world stress test demonstrated that large-scale telehealth deployment can be both clinically effective and cost-efficient, laying the groundwork for the sustained integration of virtual MDTs in routine care [21].

Telemedicine permits healthcare providers from various disciplines to work efficiently together, regardless of whether they are geographically separated. Technology plays a critical role in telemedicine, providing the framework for virtual consultations, diagnostics, and patient monitoring [17]. Video conferencing solutions used in telehealth appointments provide high-quality audio and video capabilities that aid in accurate diagnosis and successful communication. Furthermore, secure platforms are critical for maintaining patient confidentiality and complying with legislation, like HIPAA in the United States and GDPR in Europe [22]. Integration with electronic health records improves workflow efficiency, allowing physicians to access current patient data in real time [23].

Telemedicine in multidisciplinary care necessitates careful planning and adaptation to local environments, especially in terms of infrastructure and cultural factors. One of the most difficult aspects of establishing telemedicine is providing fair access to technology and healthcare services, particularly in low-resource areas [24]. Building a strong telemedicine infrastructure entails establishing dependable Internet connectivity, purchasing appropriate technology, and training both professionals and patients on how to use the technology [25]. According to studies, comprehensive training programs improve the likelihood of effective telemedicine integration, allowing physicians to feel more secure in utilizing the technology and allowing patients to participate more actively in their care [26]. Pilot programs can help examine the feasibility and scalability of telemedicine services before they are widely deployed [27]. Project ECHO is a structured tele-mentoring model that turns virtual MDTs into ongoing learning communities. A core group of specialists hosts regular videoconference clinics. Community-based providers at remote spoke sites join each session, hear a brief expert lecture, and then present real patient cases for collaborative discussion [28]. The ECHO model has been adopted by multiple academic centers and community hospitals to create virtual MDTs across various specialties. The University of Cincinnati’s Epilepsy/Neurology ECHO Pilot ran 20 monthly, one-hour TeleECHO clinics, with over 97% of participants reporting increased confidence in epilepsy management [29]. Similarly, the “Parkinson ECHO” Feasibility Study provided tele-mentoring on Parkinson’s diagnosis and treatment [30]. Additionally, Pediatric Palliative Care ECHO ran for two years and it achieved high acceptability, statistically significant gains in knowledge and self-efficacy and most participants reported positive practice changes at six months [31].

Multidisciplinary teams should establish a structured framework for continuous evaluation of telemedicine’s influence on care delivery. Institutions should also encourage regular feedback loops between patients and providers to promote continual progress [32].

Research emphasizes the need to create telemedicine systems that prioritize patient requirements, such as reducing technical challenges and enhancing user interface design. Patients should feel at ease utilizing the technology and confident that the virtual format will not jeopardize their treatment [33].

Studies have also given general recommendations for the implementation of virtual MDT. To transition from a traditional to a virtual multidisciplinary consultation framework, the “Model for Improvement” methodology should be used, which consists of four steps, namely plan, do, study, and act. During planning, organizers should work with IT professionals to evaluate expenses, infrastructure, security, and practicality, followed by trial sessions and surveys. For implementation, meeting times, case preparation, and effective communication should be prioritized. Screen-sharing of computer tomography (CT) and magnetic resonance imaging (MRI) studies can allow all participants, surgeons, oncologists, pathologists, and other specialists, to view identical images simultaneously, helping to facilitate a shared understanding of disease extent and treatment response [34]. Performance can be assessed through systematic data collection, linking patient information to electronic medical records, and evaluating metrics, like participation rates and guideline adherence [35].

Virtual MDTs can be highly cost-effective by lowering patient and clinician travel expenses and leveraging economies of scale once a minimum caseload is met. In a review, cost-minimization studies show that over half of virtual MDT services run cheaper than in-person care, and cost-effectiveness analyses often find them “dominant” (lower cost with equal or better outcomes). Only two studies showed higher telehealth costs, attributable to added on-site clinician salaries in hybrid models. In 25 cost–utility analyses evaluating health-related quality of life (QALYs), eight (32%) interventions were both cost-saving and QALY-gaining. The remaining studies delivered modest QALY gains at higher costs, highlighting the need to assess the clinical meaningfulness of these marginal improvements [36].

Finally, incorporating telemedicine into multidisciplinary care has the potential to significantly improve healthcare delivery, particularly for patients in remote or underserved areas. To achieve success, it must be carefully considered in terms of technology, design, oversight, and implementation [37,38]. Adopting telemedicine literature guidelines can help guide the creation of reliable, patient-centered, and effective virtual healthcare systems.

## 4. Virtual Multidisciplinary Teams Across Medical Specialties

### 4.1. Oncology

The interdisciplinary approach is critical in oncology since treatment options must be personalized to individual patients based on tumor characteristics and stage. Traditional multidisciplinary tumor boards are essential for ensuring coordinated, evidence-based cancer treatment programs [39]. During the COVID-19 epidemic, virtual tumor boards allowed for the continuation of these essential interdisciplinary approaches [40]. Healthcare professionals were able to interact remotely, evaluate imaging and pathology results, and identify effective treatment options while limiting patient exposure to the virus thanks to secure teleconferencing technology [40,41]. Research has demonstrated that telemedicine maintains the same degree of decision-making quality as in-person meetings while increasing efficiency and decreasing logistical restrictions [41,42]. Telemedicine-based multidisciplinary teams have been used to evaluate, manage, and follow up on a variety of cancers, including pulmonary [43], hepatic [44], urologic [45], musculoskeletal [46], head and neck [47,48], breast [49], neuro-oncologic [50], and prostate cancers [51]. Telemedicine studies have also demonstrated increased patient satisfaction, particularly due to improved accessibility, reduced travel burden, and streamlined multidisciplinary care [45,52].

The comparison of Internet-based multidisciplinary team management of genitourinary cancers in China prior to and following the COVID-19 epidemic shows a considerable increase in both the number of patients receiving remote consultations and the platform’s overall impact. Patients reported an improvement in diagnosis and care, with a higher percentage reaching affirmative results following consultations during the epidemic. The transition to telemedicine not only secured patient care during a crisis, but also improved educational outreach and coordination among medical institutes across the country [53].

Numerous cancer centers have transitioned to a fully virtual tumor board format. Dharmarajan et al. documented their experience implementing a virtual multidisciplinary tumor board (MDTB) during the COVID-19 pandemic. Their findings indicated that 57.9% of medical doctors favored the virtual MDTB over the traditional in-person format. Additionally, 78% expressed a preference for continuing virtual meetings, citing increased accessibility and greater participation from external physicians. This virtual MDTB used Microsoft Teams, and a group of medical doctors and trainees participated, while radiologic images were seamlessly displayed to all attendees via the screen-sharing feature at the outset [35]. A panel of colorectal surgery experts opposed using telemedicine for initial colorectal consultations or surgical decisions, favoring in-person evaluations for proper treatment planning. A prior consensus in proctology also found low acceptance of telemedicine for most conditions due to cancer misdiagnosis concerns, except for pilonidal disease and ostomy care. However, teleconsultation was considered suitable for screening, pre-hospitalization consultation, and follow-up [54].

Another study implemented a hybrid telemedicine model to guarantee continued geriatric oncology care during the COVID-19 pandemic. Key adaptations included pre-clinic assessments via telemedicine, virtual multidisciplinary team discussions, and a hybrid consultation model where one doctor was present in person while the other joined remotely. Additionally, nurses conducted scheduled follow-up calls to monitor treatment-related toxicities and geriatric syndromes. Telemedicine reduced hospital visit times from four hours to two and a half hours, and 84.8% of patients expressed satisfaction with the model. Quality of life assessments showed that 33.9% of patients reported improvements, while 58.1% maintained their global health status [55]. In a case-description of a woman with recurrent dermatofibrosarcoma, a radiologist drove the imaging review, with the use of screen-shared serial MRI and CT scans via Zoom and the NAVIFY Tumor Board, in order to help the surgeon, oncologist, and pathologist reach a fully informed consensus on next steps [56].

With the start of the COVID-19 epidemic, a hospital providing palliative care for advanced cancer patients switched to telemedicine, allowing for ongoing care via remote assessments, video consultations, phone calls, and texting. This change allowed the program to provide continuity of care to patients with advanced cancer while reducing the risk of contagion for both patients and healthcare staff [57].

Crow et al. describe a multidisciplinary liver tumor board enhancing access to specialized care for hepatocellular carcinoma patients, particularly through virtual consultations that overcome geographic and logistical barriers [58]. A single-day multidisciplinary model facilitated rapid liver transplant evaluations, improved coordination among specialists, and integrated early palliative care, ultimately streamlining treatment decisions and improving patient outcomes. However, challenges included extended consultation times, logistical difficulties in coordinating clinician schedules, and technological barriers for some patients. Insurance restrictions on multispecialty and telemedicine visits have also led some institutions to revert to in-person models. Despite these limitations, the hybrid approach remains a valuable strategy for improving access to comprehensive liver cancer care [58].

Telemedicine approaches allow for the real-time telemonitoring of patients at home and can partially replace in-person visits with teleconferences. The Outpatient Oncological Neurosurgery program was developed and implemented in a tertiary care hospital to offer outpatient neurosurgery for neuro-oncological patients. The protocol was piloted with 10 procedures and included dedicated training, a patient information guide, and postoperative telemedicine follow-up. Despite barriers, like healthcare provider preferences, patient reluctance, and geographic limitations, the program succeeded with the support of a hospital-at-home unit and telemedicine strategies [59].

A multidisciplinary team developed an integrated model for managing immune-related adverse events (irAEs) in oncology patients. Specialists from multiple disciplines—including oncology, pulmonology, immunology, endocrinology, gastroenterology, cardiology, nephrology, neurology, and internal medicine—collaborated to standardize patient care and streamline the diagnostic and treatment process. They developed a telemedicine-based workflow using a shared platform, allowing real-time consultation and intervention while minimizing unnecessary hospital transfers. Additionally, they created educational materials, formulated trust-wide guidelines, and implemented a structured assessment system to ensure rapid recognition and management of irAEs. The pilot phase has established proactive surveillance, integrating patient-reported symptoms and laboratory findings to enable timely specialist intervention via teleconsultation, reducing hospital admissions and treatment discontinuation. The second phase focuses on refining processes based on outcome analysis and defining key performance indicators for care efficiency. Finally, the third phase aims to expand the model across the entire region [60].

Telemedicine also addresses the gap in specialized care for low-income countries which often lack the necessary expertise and resources, by allowing remote expert consultations, which improve diagnosis, treatment planning, and patient outcomes. One study reviewed the impact of a Global Neuroblastoma Network tumor board accessible to low- and middle-income countries caring for pediatric patients. Despite challenges, such as pathology classification, all participants found the tumor board to be helpful, with 70% adjusting their care plans based on the discussions [61]. Furthermore, a real-time 3D Telemedicine system has demonstrated its potential to revolutionize surgical collaborations in low-income countries by enabling international multidisciplinary teams to provide expert consultations remotely, improving surgical planning, patient understanding, and postoperative care [62].

Another study employed a virtual-hybrid strategy to create and assess a cardio-oncology clinic specializing in cardiovascular problems among cancer survivors. The researchers used virtual consultations and multidisciplinary teamwork to improve patient care during and after the COVID-19 epidemic. The clinic was created using concepts from pre-existing cardio-oncology programs, networking with experts, and evaluating institutional resources [63].

### 4.2. Cardiology

Telemedicine has transformed multidisciplinary collaboration in cardiology, allowing coordination between cardiologists, cardiac surgeons, and related specialists. Wearable technologies and home-based diagnostics improve patient care by delivering real-time cardiac data, allowing for proactive decision making. Studies regarding remote case management of heart failure cases have agreed that patient education and self-management are essential components of multidisciplinary care, as is a comprehensive strategy to effectively manage comorbid diseases [64]. Managing heart failure, type 2 diabetes mellitus, and chronic kidney disease as a unified syndrome requires multidisciplinary teams, integrated care models, and telemedicine solutions [65]. In a pilot study of patients with congestive heart failure (HF) and left ventricular ejection fraction > 40%, telemedicine was associated with a lower risk of non-fatal acute HF events along with a lower risk of hospitalization at the six months follow-up [66].

Telemedicine supports vitamin K antagonist (VKA) monitoring by extending international normalized ratio (INR) testing intervals and identifying candidates for direct oral anticoagulants (DOAC) switching [67]. eHealth-based management is more cost-effective than traditional care, reducing complications and hospitalizations, particularly for anticoagulation therapy [68]. It also improved adherence among DOAC users during the COVID-19 pandemic [69]. Overall, telemedicine strengthens collaboration among physicians, nurses, and pharmacists, also improving atrial fibrillation management [56]. An institution in Italy rapidly transitioned to a telemedicine-based multidisciplinary unit (tele-MDU) for myocarditis management during the COVID-19 pandemic. Using HealthMeeting^®^, specialists collaborated remotely for diagnostics, follow-ups, and treatment adjustments. This strategy made it possible for smooth inpatient-to-outpatient transitions, fast transmission of endomyocardial biopsy results, and early discharge with ongoing monitoring. Effective care was guaranteed by hybrid approaches, which included video conversations and in-person visits when required [70].

A multidisciplinary team designed a telerehabilitation program for patients awaiting elective cardiac surgery. The intervention group had a significantly lower incidence of major adverse cardiovascular events at one year (16.8% vs. 25.5%), primarily due to reduced hospitalizations. The greatest benefit was observed in surgical patients. Telerehabilitation also improved modifiable risk factors, including smoking, pulmonary risk, and depression, but had no impact on hospital stays or postoperative complications [71]. HerzMobil Tirol PreOp has also been designed as a multidisciplinary telemedicine project with the role of optimizing high-risk heart failure patients before elective cardiac surgery. The initiative aimed to reduce perioperative complications through telemedicine-assisted care [72].

### 4.3. Neurology

Despite the advantages of a multidisciplinary team approach for long-term neurological disorders, availability remains a barrier because neurological care is frequently centered in large tertiary facilities, leaving other regions underserved [73]. Telemedicine is increasingly being used in neurology for distant evaluation and treatment of a variety of disorders. Telestroke services allow for faster assessment and thrombolysis decision making in acute ischemic stroke patients [74]. For epileptic and non-epileptic seizures, it allows for seizure monitoring, medication titration, and patient counseling [75]. Telemedicine can help with remote motor assessments, cognitive evaluations, disease progression, fall prevention, and therapy changes for Parkinson’s disease [76,77,78], Alzheimer’s disease, and other neurodegenerative disease [79,80]. Remote monitoring also helps with functional assessments and respiratory and medication adjustments in neuromuscular illnesses, like amyotrophic lateral sclerosis (ALS) [81]. Telemedicine can also incorporate psychoeducation and peer support for patients with psychosis [79,82]. Although telehealth and telestroke have been explored and implemented in recent years, significant advancements are still needed to optimize telemedicine for neurological diseases. A study compared the F@ce 2.0 programme, a person-centered rehabilitation intervention for stroke patients, to traditional rehabilitation. The trial involved stroke patients, their significant others, and interdisciplinary rehabilitation teams. The intervention incorporated goal setting, problem-solving skills, and digital support via a web platform that enabled daily reminders and progress tracking. The primary goals were self-efficacy and performance in everyday tasks, while the secondary outcomes were functional independence, fatigue, mood, and life satisfaction. Qualitative and process evaluations were also carried out to assess implementation and outcomes [83]. Studies have shown through nurse-driven e-alert integration, coordinated interdisciplinary training, and in-suite tele-neurology consults, a community emergency department without on-site neurology dramatically accelerated door-to-neurologist assessment and treatment times for stroke alerts [84]. Newer research highlighted that, by leveraging web-based messaging portal alongside applications, like WhatsApp, healthcare teams in low-resource regions can tap into remote neurologists’ expertise and dispatch instant alerts to stroke response units, streamlining patient evaluation and care even without advanced teleconferencing infrastructure [85]. Additionally, by converting routine CT/MRI data into quick-to-generate 3D virtual reconstructions, neurosurgeons gained clearer views for preoperative planning, included teleradiology-enriched MDT in their discussions, and improved patient shared decision making [86].

The cost-effectiveness analysis of the virtual multidisciplinary stroke care clinic (VMSCC) service suggests that while the intervention may lead to reductions in emergency admissions and hospital stays, the additional costs incurred must be carefully considered. The economic viability of scaling up such a service depends on healthcare system priorities and funding availability [87]. The VA’s National Telestroke Program cut interhospital transfers for acute ischemic stroke by 14.4% by providing remote neurologist support. This not only improves on-site stroke care but also optimizes patient disposition and curbs unnecessary transfers, especially at lower-volume hospitals [88]. Short-term post-discharge telerehab boosts language and memory, but its impact on behavioral symptoms remains unclear [89]. A home-based tele-neurorehabilitation program was implemented using the VRRS platform, delivering daily personalized cognitive exercises (attention, memory, praxis, language, and orientation) to patients with stroke, under remote supervision by a multidisciplinary team and aided by caregivers. Post-treatment, patients showed gains in working memory and language praxis, depression scores dropped significantly, while overall quality-of-life scores remained the same [90].

A study evaluated the impact of a multidisciplinary telemedicine program on frailty in Parkinson’s disease. The telemedicine group received additional care from a neurologist, nurse, and occupational therapist between the first two visits. Frailty was significantly reduced in the telemedicine group compared to controls, alongside improvements in gait freezing, balance, gait speed, fatigue, non-motor symptoms, and quality of life [77].

During an ALS study, telemonitoring was conducted for ALS patients through video televisits involving neurologists, dieticians, psychologists, and physiotherapists, with each patient receiving an average of three visits per month per specialist. Neurological management included medication adjustments, respiratory assessments led to non-invasive ventilation initiation in two patients, and multidisciplinary follow-ups were scheduled post-pandemic, while dietary, psychological, and physiotherapy interventions focused on maintaining nutritional status, mental health stability, and functional support, with most patients expressing satisfaction with the program [91]. Another study revealed that patients were more likely to participate in non-epileptic seizures treatment when their initial neurological consultations were conducted via telemedicine. Telehealth also improved access for publicly insured and employed patients, most likely due to its greater flexibility and fewer logistical constraints. Furthermore, individuals with fewer comorbidities and less frequent seizures were more likely to use telehealth, but those with a history of suicidal thoughts were less likely to do so [75].

### 4.4. Pulmonology

For individuals with respiratory diseases, the care team may consist of doctors, nurses, physiotherapists, speech therapists, dietitians, and psychologists, along with specialists from other specialties, such as endocrinology, gastroenterology, allergology, and otolaryngology [92]. Within this field, cystic fibrosis was one of the first conditions where the value of specialist multidisciplinary teams was acknowledged. Patients treated at specialized centers showed improved lung function, body mass index (BMI), and chest and radiography scores [93].

The COMETA project is a learning program designed to teach medical professionals how to manage asthma with an emphasis on teleconsultation integration. Experts from the fields of pulmonology, allergy, general medicine, nursing, and pharmacy are involved. The project focuses on three main areas, namely medical telecare for patient follow-up, asthma control, and provider collaboration to improve the quality of care. With an emphasis on better asthma control and enhanced patient quality of life, COMETA promotes the shift to a new care model that blends in-person visits with telecare [94]. The TEAM 2.0 project aimed to update and prioritize best practices in multidisciplinary asthma care post-pandemic. A coordinating group of hospital pharmacists, pulmonologists, and allergists reviewed the literature, shared practices, and analyzed progress through regional meetings. The project successfully updated the roadmap for optimal care models for asthma patients in the post-COVID-19 era [95]. Multidisciplinary teams have also developed mHealth apps for children with asthma, designed to improve disease management by supporting medication adherence, symptom tracking, and education [96,97].

For patients with cystic fibrosis, guidelines call for quarterly multidisciplinary care. This can be difficult for people who live far away from specialized centers. Interest in remote monitoring and telemedicine has increased as a result. With new research showing the viability of telemedicine for remote spirometry, respiratory culture collection, adherence tracking, symptom assessment and tracking, and activity evaluation, patients with cystic fibrosis might transition from face-to-face clinics to multidisciplinary virtual clinics [98,99,100].

### 4.5. Orthopaedics

Telemedicine in orthopedics and trauma surgery has primarily advanced in such areas as joint arthroplasty, fracture therapy and management, and pre- and postoperative care, including teleradiology [101]. The ACTIVE trial is a multicenter, parallel randomized controlled experiment that evaluates the efficacy and economic implications of a multidisciplinary integrated care program for knee arthroplasty patients. The intervention is made up of three parts, namely the tailored ikHerstel app, goal setting with goal attainment scaling, and a case-manager referral. Patients are randomized one-to-one and stratified based on medical center, surgery type, and recovery expectations. The ikHerstel app, which includes an activity tracker and adaptive recovery recommendations, offers preoperative education, tailored recovery plans, and postoperative monitoring [102].

Another study established a telemedicine-based MDT approach in Armenia, where international and local experts collaborated to review and manage musculoskeletal sarcoma cases, significantly improving patient care and treatment outcomes [103]. The comprehensive review of Wahezi et al. focused on the adaptation of telemedicine during the COVID-19 pandemic, stressing the interdisciplinary strategy used to provide high-quality musculoskeletal and neurologic assessments. The study, which brings together experts in physical medicine and rehabilitation, orthopedics, rheumatology, neurology, and anesthesia, emphasizes the collaborative effort required to change and validate physical examination approaches for remote evaluation. This interdisciplinary consensus not only improves the feasibility of telemedicine but also assures that patients continue to receive comprehensive care despite the constraints provided by fewer in-person appointments [104].

### 4.6. Endocrinology and Metabolic Diseases

Telemedicine has been shown in studies to assist diabetes mellitus (DM) care by improving glucose parameters [105,106], increasing patient participation and provider satisfaction [107]. During the COVID-19 pandemic, clinics treating patients with type 1 DM increased telemedicine visits from <1% to 94.7% within a month, guaranteeing continuity of service despite early difficulties. Multidisciplinary teams, including diabetes educators, nutritionists, nursing staff, social service providers, and psychologists, have started to use virtual workflows, meeting patients independently or asynchronously with medical doctors [108]. One of the most common and important complications of DM, diabetic foot ulcer (DFU), has also been studied, as telemedicine allows for a multi-sectorial and interdisciplinary close follow-up. This includes monitoring and controlling the care pathway and taking early action to stop DFUs from occurring repeatedly [109].

Telemedicine has proven effective for obesity management, particularly during pandemics, and has remained integral beyond this period. Experts recommend a hybrid care model, combining telemedicine with in-person visits, to facilitate intensive management through frequent multidisciplinary team follow-ups [110]. A study on a weight management program in Australia included telephone health coaching along with multidisciplinary care. The results demonstrated that integrating telemedicine with standard care led to significant health benefits and was well-received by patients, suggesting that such telemedicine interventions can enhance obesity management programs [111]. Bariatric surgery also requires a multidisciplinary team and frequent pre- and postoperative visits. After transitioning to telehealth, a multidisciplinary clinic has reported that new patient visits declined, likely due to the pandemic’s impact on employment, insurance, and patient preference for in-person care. However, follow-up visits saw nearly a three-fold reduction in no-show rates, suggesting that existing patients were more likely to attend virtual appointments than new ones [112].

### 4.7. RE-AIM Framework

To evaluate virtual MDT interventions, we systematically applied the Reach, Effectiveness, Adoption, Implementation, and Maintenance (RE-AIM) framework [113] to a series of peer-reviewed studies that all reported clinical processes/patient-level outcomes (Table 1). We began by quantifying reach in each trial, reporting the numbers and characteristics (if available) of patients screened. Effectiveness was then established through clinical endpoints drawn from these studies. Adoption was measured at the provider and site level, documenting how many centers, specialties, and individual clinicians actually implemented the virtual MDT model, as reported in each investigation. For implementation, we extracted the detailed operational protocols, platforms used, staffing mixes, training regimens, fidelity checks, and resource inputs. Finally, maintenance was assessed by tracking the duration of both program delivery or patient follow-up within each study.

While a growing number of tele-MDT models have been used across diverse conditions, all of the published reports remain single-site, short-term demonstrations. Few extend beyond six to twelve months, and only a minority rigorously capture patient-level clinical endpoints. As a result, the studies reveal consistent signals of feasibility, high satisfaction, and promising intermediate outcomes, but leaves unanswered whether virtual MDT care can be reliably sustained over years or generalized across health systems and whether it shows durable improvements in outcomes, such as mortality, hospitalization rates, and disease progression.

### 4.8. Patient Satisfaction and Healthcare Provider Perspectives

Numerous studies have examined the opinions of both patients and clinicians regarding telemedicine, assessing its feasibility, advantages, and limitations compared to traditional face-to-face consultations (Table 2). A study reported that patients generally felt comfortable and reassured after learning that their case was discussed by an MDTB, valuing the collective expertise involved in their treatment decisions. While most patients understood and were satisfied with the recommendations, some felt they had limited involvement in the decision-making process. In terms of communication preferences, patients strongly favored in-person discussions over electronic patient portals or written letters, citing concerns about security and a lack of engagement with digital platforms. In the pilot telemedicine intervention, most patients found virtual consultations to be efficient and satisfactory, with high levels of comfort in asking questions and understanding their treatment plans. However, despite these positive experiences, half of the patients still preferred in-person visits over telemedicine, though a majority would recommend virtual consultations to other patients [44].

Patients with ALS and caregivers appreciated teleconsultations for their convenience and continuity of multidisciplinary care at home. However, they noted drawbacks, such as limited physical contact and technological challenges. Teleconsultations also fostered collaboration among the care team and professionals from the patient’s home city [81]. The majority of participants expressed a preference for continued inclusion in remote evaluation programs [114]. Patients with Huntington’s disease and their caregivers expressed overall satisfaction with telehealth, with a mean attitude score of 5.92/7. Most (88%) found it an acceptable alternative to in-person visits, valuing healthcare accessibility (6.12/7), communication with providers (6.06/7), and usability (5.72/7). Some patients (21%) reported difficulties due to age or neurocognitive issues [80]. The majority of patients in studies found the hybrid telemedicine system beneficial, with one study reporting that 84.8% of patients expressed satisfaction, citing reduced hospital visit times and improved convenience [55].

Another study reported patient satisfaction as perceived by their medical doctors, with responding physicians reporting that their patients, caretakers, colleagues, and staff were all extremely satisfied with telemedicine, with patients being the most satisfied of the four groups [115]. The convenience and efficiency of virtual visits were the most appreciated aspects reported by patients in different studies [116]. Despite positive feedback in the majority of studies, technical failures also led to the cancellation of planned consultations [117]. Two Italian centers reported that virtual care drove significantly higher patient satisfaction, especially for cost and convenience [118].

In a study evaluating parents, children, and clinicians, they all expressed positive views about the use of telemedicine in the multidisciplinary pediatric neuromuscular clinic. Parents appreciated the reduced travel time and flexibility, with over 90% reporting that their child’s issues were addressed during telemedicine appointments. Both parents and children generally found telemedicine less stressful than face-to-face appointments, although parents noted challenges, such as managing their child’s behavior during sessions. Clinicians valued the improvements in patient attendance and time management, though they expressed concern about providing high-quality care via telemedicine [119].

Rajasekaran et al. [46] investigated clinicians’ views concerning virtual MDT meetings, which were mostly positive. Most doctors believed that virtual MDTs provided adequate interaction (72.2%), maintained decision-making quality, and secured access to patient data (86%). The satisfaction levels were mixed, with 36.1% highly satisfied, 38.9% moderately satisfied, and only 13.9% dissatisfied. Looking ahead, 75% believed virtual MDTs supported cancer care standards, 77.8% saw them as the future of oncology, and 91.7% saw the potential for worldwide case discussions. In another study, clinicians expressed varying opinions on the utilization of virtual MDT meetings in cancer care. While telemedicine was widely accepted for patient management, only 26% of respondents believed that an in-person visit was required for MDT discussions. However, certain questions persisted about the effectiveness of virtual platforms for complicated case discussions and collaborative decision making. Despite these concerns, the majority of doctors stated a desire to maintain or even increase telehealth utilization [115]. On the contrary, clinicians in the INSPIRERS project expressed concerns about inadequate institutional support for telemedicine, with most relying on telephonic consultations as the main alternative to in-person visits and only a fifth using video consultations [118]. A team of radiologists reacted positively to moving MDTs online: 69% approved immediately, and 78% felt their role stayed the same. While 34% found virtual MDTs equivalent to in-person and 31% found them better, 34% found them worse. Technical/connectivity issues were the chief drawback (67%), and 29% noted suboptimal image viewing. A majority (61%) supported a future hybrid model [120].

Some participants also appreciated how easy they found it to access and share clinical information during sessions. However, concerns were expressed about the impact on team cohesion, communication, and engagement. The lack of in-person interactions created feelings of isolation, and several members struggled to form solid professional ties [61]. Other studies reported that only 40% of doctors believed these consultations reduced their workload, while even fewer of them (36%/33%) believed that they helped significantly with psychological or social issues [121].

Another study included multidisciplinary virtual visits for patients with DM [105]. Clinicians had mixed opinions about video visits for diabetes care. While some appreciated the convenience and flexibility, others found them less effective due to the lack of physical exams, difficulty sharing glucose data, and increased responsibility for self-monitoring. Many patients who were highly engaged and prepared for the visits benefited the most, while those with technological or health literacy barriers struggled. Some patients preferred face-to-face visits for more comprehensive care, especially for initial consultations and complex cases [105].

**Table 2:** Common disadvantages of virtual multidisciplinary consultations across different specialties.

Study, Year	Medical Specialty	Reported Disadvantages
Barrios et al., 2022 [122]	Cardiology	Symptomatic patients, lack of specific training/infrastructure, sensory disabilities
Carroll et al., 2022 [119]	Neurology	Lack of physical “hands on” assessment
Chavarri-Guerra et al., 2021 [57]	Oncology	Technological barriers, privacy issues
Chen et al., 2022 [55]	Oncology	Impaired hearing and vision, cognitive impairment, limited digital literacy
Crow et al., 2023 [58]	Oncology	Logistical challenges, technological barriers, insurance limitations
Dharmarajan et al., 2020 [35]	Oncology	Poor sound quality, unstable connections, and screen-sharing limitations, lack of personal interaction
Fidelix et al., 2023 [81]	Neurology	Lack of physical contact, difficulties with technology
Groothuizen et al., 2023 [123]	Oncology	IT issues, reduced team cohesion and informal communication, risk of disengagement
Mora et al., 2022 [59]	Oncology	Lack of experience, lack of specific funding
Zupa et al., 2025 [124]	Endocrinology	Lack of clinical examination and data

## 5. Conclusions

Virtual multidisciplinary teams in healthcare became critical during the COVID-19 pandemic, allowing for continuing patient treatment via telemedicine. The use of digital tools promoted virtual communication among specialists, increasing efficiency and accessibility, particularly for patients in distant regions. While virtual MDTs have shown benefits, such as shorter travel times and higher patient satisfaction, difficulties, such as technology obstacles and team cohesion, remain.

## Figures and Tables

**Table 1:** RE-AIM framework.

Study	Reach	Effectiveness	Adoption	Implementation	Maintenance
Chen et al. [55]	690 patients	Reports high patient satisfaction (84.8%), reduced clinic time (4 h → 2.5 h), and quality of life maintained/improved in ~92%	Fully describes the multidisciplinary team (geriatricians, oncologists, nurses, allied health) and tele-workflow	Detailed protocol: pre-clinic tele-meeting, hybrid onsite/remote consults, and nurse follow-up calls, with fidelity measures (clinic time saved, % receiving interventions)	Sustained through intermittent pandemic restrictions and resumed service over a full year, yet no data on program continuation
Fidelix et al. [81]	71 patients; 46 (64.7%) participated	High patient/caregiver satisfaction and continuity of multidisciplinary care reported, with key issues (respiratory, meds) addressed	Full MDT (neurology, physical therapy, speech, diet, psychology) co-opted the platform; 65% of eligible patients engaged	Detailed protocol: pre-visit screening, shared virtual room, structured satisfaction surveys	One-year follow-up
Jiménez-Marrero et al. [66]	116 of 178 original iCOR patients (65%), all drawn from a single center’s post-discharge CHF program	Tele-CHF cut acute non-fatal HF events by two-thirds and reduced HF- and CV hospitalizations, with lower costs	Implemented by the single center’s heart-failure team, comprising cardiologists, specialist nurses, and IT support	Integrated videoconference and daily bio-monitoring into existing nurse-led CHF pathway, with standard protocols; reliance on custom IT platform and specialist nursing time	Six-month follow-up
Eggebrecht et al. [68]	705 patients in the experimental group and 1490 patients in the control group drawn from a large German database	Coagulation intervention cut all-cause admissions from 68.7 to 23.4/100 py, halved OAC-related stays, and saved costs overall	Coagulation model already embedded in practice and offered to all eligible VKA patients	Detailed staffing, eHealth platform, and workflows described, requires specialist nurse time, IT support and therapy protocols	One-year follow up
Peretto et al. [70]	29 new myocarditis cases; 115 of the existing cohort	Preserved diagnostic/treatment timelines, no COVID-19-related myocarditis or opportunistic infections, 6 ± 2 days saved in length-of-stay, zero loss to follow-up, and compliance maintained	HealthMeeting^®^ platform; referral physicians handled 94% of visits; adoption was universal within the unit	Detailed workflows for inpatient early discharge, outpatient hybrid visit models (video/phone/email/in-person), referral-physician triage	Four-month follow up
Scheenstra et al. [71]	394 of all eligible elective cardiac surgery/procedure patients across several referral centers	One-year major adverse cardiovascular events decreased from 25.5% to 16.8% driven by fewer postop events; modest quality of life gains; no impact on preop events	Three collaborating centers, full team (seven surgeons, physiotherapists, dietitians, and psychologists)	Detailed: web platform integration, risk-factor screening, 5 modular interventions tailored by case managers, blended delivery (video/phone/email), stratified randomization, rigorous follow-up protocol	One-year postop follow-up
Watson et al. [75]	104 patients in a single center	Increased initial treatment uptake	Neurology + behavioral health)	Detailed protocols: three scheduling attempts, hybrid visit inclusion, rigorous cohort definitions, and detailed demographic analyses	Only during the study window
Garcia-Bustillo et al. [77]	50 patients at one movement-disorders center	Significant and sustained reductions in frailty at 4 m and 8 m, plus improvements in balance, gait, non-motor burden, and quality of life	Delivered by a multidisciplinary team (neurologists, nurses, physical therapy, psychologist, and engineers)	Protocol, home setup, and 45–60 min real-time sessions are clearly described, and wearable monitoring used	Eight-month follow-up
Lam et al. [87]	256 stroke survivors across 10 public hospitals	Modest reductions in emergency admissions and hospital days, but no statistical testing was reported	Delivered in 10 sites, multicenter uptake	Protocol: intervention components (video calls, BP monitors, platform access)	Six-month follow-up
De Marchi et al. [9]	19 of 91 patients scheduled for multidisciplinary care	Reported clinical measures (ALSFRS-R, BMI, HADS, Barthel) showed stability or modest improvements	Four disciplines (neurology, dietetics, psychology, and physio) and patients adopted the teleplatform	Protocol: intervention components (platform, visit frequency, multidisciplinary roles)	Two-month follow up
Hsia et al. [96]	39 patients	Significant within-subject gains in asthma control, knowledge, quality of life, and reductions in emergency department visits and prednisone use over three visits	One care team; all completers engaged with the app across three visits	Intervention components, session lengths, and assessment schedules were described	Eight-month follow-up
Papyan et al. [103]	84 pediatric sarcoma patients	Qualitative improvements in diagnostic accuracy and treatment approaches	Fully integrated into four multidisciplinary working groups	Described meeting cadence, case volumes, and telemedicine platform use	Study over 4 years
Sanal et al. [105]	50 patients	MDT-assisted group showed significant reductions in HbA1c, mean glucose, hypo- and hyperglycemia episodes, and reported better psychosocial outlook	A single center using the DTMS^®^ platform and one multidisciplinary team	Described trimonthly carb-counting training and continuous MDT support	Outcomes reported only over the study period; no follow-up data
Driscoll et al. [111]	41 control and 39 intervention patients	Intervention group lost nearly twice the weight of controls (−12.6 kg vs. −6.7 kg) and showed a significant HbA1c reduction (−0.7% vs. −0.2%,), with favorable but non-significant trends in liver stiffness and enzymes	Implemented at a single tertiary program	Coaching protocol (13 calls over 5 months), training of coaches by dietitian, and integration with existing MDT workflow	Six-months post-baseline

## Data Availability

All data can be requested from the corresponding author.
